# In vitro migration and proliferation (“wound healing”) potential of mesenchymal stromal cells generated from human CD271^+^ bone marrow mononuclear cells

**DOI:** 10.1186/s12967-015-0676-9

**Published:** 2015-09-25

**Authors:** Hatixhe Latifi-Pupovci, Zyrafete Kuçi, Sibylle Wehner, Halvard Bönig, Ralf Lieberz, Thomas Klingebiel, Peter Bader, Selim Kuçi

**Affiliations:** Department for Children and Adolescents, Division for Stem Cell Transplantation and Immunology, Goethe University Hospital, Frankfurt am Main, Germany; Institute of Transfusion Medicine and German Red Cross Blood Center Frankfurt, Frankfurt am Main, Germany; Institute of Pathology, Goethe University Hospital, Frankfurt am Main, Germany

**Keywords:** Bone marrow, MSC-subsets, Wound healing potential

## Abstract

**Background:**

Emerging evidence indicates that mesenchymal stromal cells (MSCs) isolated from different tissue sources may be used in vivo as tissue restorative agents. To date, there is no evidence, however, on migration and proliferation (“wound healing”) potential of different subsets of MSCs. The main goal of this study was therefore to compare the in vitro “wound healing” capacity of MSCs generated from positively selected CD271^+^ bone marrow mononuclear cells (CD271-MSCs) and MSCs generated by plastic adherence (PA-MSCs).

**Methods:**

The in vitro model of wound healing (CytoSelect™ 24-Well Wound Healing Assay) was used in order to compare the migration and proliferation potential of CD271-MSCs and PA-MSCs of passage 2 and 4 cultured in presence or absence of growth factors or cytokines.

**Results:**

CD271-MSCs of both passages when compared to PA-MSCs demonstrated a significantly higher potential to close the wound 12 and 24 h after initiation of the wound healing assay (P < 0.003 and P < 0.002, respectively). Noteworthy, the migration capacity of PA-MSCs of second passage was significantly improved after stimulation with FGF-2 (P < 0.02), PDGF-BB (P < 0.006), MCP-1 (P < 0.002) and IL-6 (P < 0.03), whereas only TGF-β enhanced significantly migration process of PA-MSCs of P4 12 h after the treatment (P < 0.02). Interestingly, treatment of CD271-MSCs of both passages with growth factors or cytokines did not affect their migratory potential.

**Conclusions:**

Our in vitro data provide the first evidence that CD271-MSCs are significantly more potent in “wound healing” than their counterparts PA-MSCs.

## Background

Mesenchymal stromal cells (MSCs) are non-hematopoietic multipotent cells which can be derived from bone marrow mononuclear cells [1], adipose tissue [2, 3] or other tissues such as fetal liver, lungs, spleen [4, 5], amniotic fluid [6], cord blood [7], cord [8, 9], placenta [10, 11], endometrium [12] and dental pulp [13]. MSCs can be generated either by plastic adherence of progenitor cells for MSCs (PA-MSCs) or by a positive selection with antibodies against cell surface antigens expressed by MSC-progenitor cells (D7-FIB, CD271, SSEA-4, GD2 and frizzled-9) [14–18]. CD271 (Low-affinity nerve growth factor receptor) antigen is present on human bone marrow cells and potentially defines a mesenchymal stromal cell (MSC) precursor subpopulation. Abundant evidence suggest that MSCs, isolated from different tissue sources, confer benefits in vivo as tissue restorative agents [19]. Wound healing is a complex process that requires the coordinated interplay of extra cellular matrix, growth factors, and cells. The presence of MSCs in normal skin and their critical role in wound healing suggests that the application of exogenous MSCs is a promising strategy to treat non-healing wounds resulting from trauma, diabetes, vascular insufficiency, and numerous other conditions [20]. Bone marrow-derived mesenchymal stem cells (BM-MSCs) promote the healing of diabetic wounds due to increased re-epithelialization, cellularity, and angiogenesis [21, 22]. These cells may participate directly in wound closure through progenitor cell proliferation and differentiation, as well as production of extracellular matrix (ECM) [23]. Several studies have shown that BM-MSCs secrete a variety of cytokines and growth factors which are known to enhance normal wound healing [24–27] including fibroblast growth factor-2 (FGF-2), epidermal growth factor (EGF), platelet-derived growth factor (PDGF), transforming growth factor beta (TGF-β) [28, 29], hepatocyte growth factor (HGF), interleukin 6 (IL-6), interleukin 8 (IL-8) [21] vascular endothelial growth factor (VEGF), stromal cell-derived factor (SDF) [27] and insulin-like growth factor-1 (IGF-1) [30–32]. Despite several in vivo studies which demonstrated the impact of MSCs on wound healing [33, 34], there is so far no evidence on wound healing potential of different subsets of mesenchymal stromal cells. In this in vitro study we focused on migration and proliferation (“wound healing”) capacity of MSCs generated from CD271^+^ bone marrow mononuclear cells (CD271-MSCs) and compared it to MSCs generated by plastic adherence (PA-MSCs). In addition, we evaluated the effect of different growth factors and cytokines in migration and proliferation potential of these cells.

## Methods

### Isolation of bone marrow mononuclear cells (BM-MNCs)

Bone marrow aspirates were taken from the iliac crest of 4 male healthy donors (age: 18–22 years) after informed consent and under a protocol approved by the University of Frankfurt Institutional Review Board. After dilution 1:2 in PBS, aspirates were centrifuged in 1.073 g/ml Ficoll density gradient in 700×*g* for 30 min. The enriched cells were collected from the interface, washed twice with PBS (PAA Laboratories GmbH, Austria) and centrifuged at 400×*g* for 10 min. A defined number of isolated BM-MNCs were used for generation of PA-MSCs whereas the majority of them were used for enrichment of CD271^+^ cells.

### Generation of CD271-MSCs

CD271^+^ bone marrow mononuclear cells were isolated immune-magnetically using the MSC Research Tool Box–CD271 (LNGFR)-APC (Miltenyi Biotec GmbH), according to the manufacturer’s instructions. Highly purified bone marrow CD271^+^ mononuclear cells (1.25 × 10^5^/cm^2^) were seeded in T25 (25 cm^2^) culture flasks with vent caps in 6 ml DMEM low-glucose supplemented with 10 % MSC-qualified fetal bovine serum (FBS) (GIBCO/Invitrogen, Darmstadt). The medium was changed after 7 days and later on every third day until the cells reached the confluence 70–80 % (10–14 day). MSCs generated in this way are referred to as CD271-MSCs throughout the manuscript. After this step the whole procedure was the same as for generation of PA-MSCs.

### Generation of PA-MSCs

To generate PA-MSCs, BM-MNCs were cultured in DMEM low-glucose supplemented with 10 % MSC-qualified FBS. The cells were maintained at 37 °C in 95 % humidified atmosphere of 5 % CO_2_ for 72 h. Thereafter, the nonadherent cells were removed and fresh medium was added and changed every 2 or 3 days. The adherent spindle-shaped cells were further cultured for 10–14 days until the cells reached about 70–80 % confluence. During this time the medium was changed every 3 days. To detach the MSCs the medium was removed and the cells were washed once with PBS. The cells were detached by exposure to trypsin TrypLE (Invitrogen) for 6 min at 37 °C, followed by tapping the dishes and the addition of culture medium. The cells were centrifuged then resuspended with medium and plated at a density of 2 × 10^3^ MSCs/cm^2^. During culture the medium was changed every 3 days, and when the cells were confluent they were passaged. The cells were passaged three times, and cells from the second and fourth passage were used for experiment.

### Colony forming unit-fibroblast assay and expansion potential of CD271-MSCs

To assess the clonogenic potential of positively selected CD271^+^ cells and BM-MNC, the CFU-F assay was performed in 25 cm^2^ tissue culture flasks. For this purpose, 2.5 × 10^5^ BM-MNC/25 cm^2^, and 2.5 × 10^4^ cells/25 cm^2^ from the CD271-positive fraction were cultured for 14 days. Colonies were stained with Giemsa solution (Merck, Darmstadt, Germany) and counted.

### Immunophenotyping of CD271-MSCs and PA-MSCs

CD271-MSC and PA-MSC of different passages (from passage 1 to passage 4) were stained with fluorochrome-conjugated mouse anti-human antibodies against following antigens CD73, CD90, CD105, CD146, CD44, CD29, CD166, CD45, CD34 and CD14 and HLA-Class I and HLA Class II molecules and incubated at 4 °C for 30 min. After two wash steps with PBS + 0.2 % BSA the stained cells were analyzed on a FACSCalibur (Becton–Dickinson) equipped with Macintosh software for data analysis (CellQuest).

### Trilineage differentiation of MSCs

To induce differentiation of MSCs, specific medium was added to the cells according to the manufacturer’s instructions. Adipogenic differentiation was induced by NH Adipo Diff Medium (Miltenyi Biotec, Bergisch Gladbach). Osteogenic differentiation was achieved by NH OsteoDiff Medium (Miltenyi Biotec, Bergisch Gladbach), whereas chondrogenic differentiation was induced by NH ChondroDiff Medium (Miltenyi Biotec, Bergisch Gladbach). Each specific differentiation medium was changed every 2–3 days. Confirmation of differentiation of the cells to adipocytes, osteocytes and chondrocytes were performed by staining with Oil Red O staining solution, SIGMA FAST™ BCIP/NBT (5-bromo-4-chloro-3-indolyl phosphate/nitro blue tetrazolium) tablets and Alcian blue-solution, respectively.

### Wound healing assay

The wound healing process entails the migration and proliferation of different cells, including the MSCs. To compare the wound healing potential of CD271-MSCs and PA-MSCs, the second and fourth passage MSCs were cultured in DMEM containing 1 % FBS. To analyze the wound healing capacity, we used an in vitro model of wound healing (CytoSelect™ 24-Well Wound Healing Assay) from the Cell Biolabs company (BIOCAT GmbH, Heidelberg, Germany). The CytoSelect™ 24-well Wound Healing Assay Kit consists of 2 × 24-well plates each containing 12 proprietary treated plastic inserts. The inserts create a wound field with a defined gap of 0.9 mm for measuring the migration and proliferation rate of cells. In order to precisely find the same position at making the photographs, the center of each well was labeled before adding the cells.

Briefly, CD271-MSCs and PA-MSCs cell suspension containing 2 × 10^5^ MSCs/ml DMEM with 1 % FBS was prepared. For optimal cell dispersion, 250 µL of cell suspension was added on each side of the insert by carefully inserting the pipette tip through the open end of the insert (1 × 10^5^/well/500 µL). Cells were cultured until they formed a monolayer around the insert (24 h). Next day, the inserts were removed from the wells, leaving a precise 0.9 mm open “wound field” between the cells. To assess how the “wound field” is affected by the cells itself, the wells were washed twice with PBS and then fulfilled with 500 µL of prepared medium. In addition, to see the effect of CD271-MSCs and PA-MSCs on wound closure in the presence of different growth factors (GFs) or cytokines, the wells were washed twice with PBS and then fulfilled with 500 µL of prepared medium with GFs/Cytokines (Table 1). In order to analyse migration capacity and not proliferation of the cells, the first photographs of the wounded area were taken at 0 h, after 6 and after 12 h. After that period the cells were monitored for proliferation and photographs were made after 24 h. To measure the wound closure, we set a rectangle with a defined surface area and passed that on all photos of the other time points.

**Table 1:** Growth factors and cytokines assessed for their effect on wound healing potential of MSCs

Growth factors and cytokines	Final concentration	Manufacturer
Human EGF	1 ng/ml	Pepro Tech [GmbH-Hamburg, Germany]
Human TGF-β	5 ng/ml	#
Human HGF	5 ng/ml	#
Human FGF-basic	20 ng/ml	#
Human SDF-1α	20 ng/ml	#
Human MCP-1 [MCAF]	20 ng/ml	#
Human IL-8	20 ng/ml	#
Human IL-6	10 ng/ml	#
PDGF-BB	20 ng/ml	#
Rabbit anti-human PDGF-basic	2 µg/ml	#

Estimation of percent closure was made after determination of the surface area of defined wound area (total surface area) and determination of the surface area of the migrated cells into the wounded area (migrated cell surface area): Total surface area = 0.9 mm × length; Migrated cell surface area = Length of cell migration (mm) × 2 × length, therefore:

$${\text{Percent closure }}\left( \% \right) = {\text{Migrated cell surface area}}/{\text{Total surface area }} \times { 1}00$$

Photographs were made with microscope Olympus IX71, whereas measurements were performed using Imaging software CellSens from Olympus manufacturer (Olympus, Hamburg, Germany).

### Effect of some growth factors and cytokines in “wound healing” capacity of MSCs

To assess how different growth factors and cytokines affect migration capacity we compared the effects of following 10 growth factors/cytokines on migratory activity of CD271-MSCs and PA-MSCs: epidermal growth factor (EGF), transforming growth factor beta (TGFβ), hepatocyte growth factor (HGF), fibroblast growth factor 2 (FGF-2), stromal-derived growth factor-1α (SDF-1α), monocyte chemotactic protein-1 (MCP-1), interleukin 8 (IL-8), interleukin 6 (IL-6) and platelet-derived growth factor BB (PDGF-BB). To get additional insights into possible mechanisms involved in the migratory potential of MSCs we used antibodies against one of the key players in this process, platelet-derived growth factor BB (Ab-PDGF- BB) (Table 1).

### Statistical analysis

Data were analyzed by using standard statistical software (GraphPad Prism Software, San Diego, CA, USA). Results are expressed as mean value ± standard deviation. The statistical significance of values between two groups was evaluated by Student’s *t* test. Differences were considered significant when the P-value was 0.05 or less.

## Results and discussion

### Phenotype and trilineage differentiation potential of CD271-MSCs and PA-MSCs

**Fig. 1:**
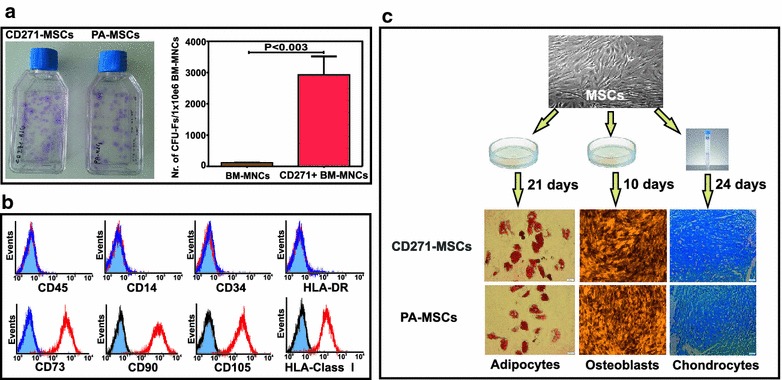
Phenotype and trilineage potential of CD271-MSCs. **a** Potential to generate CFU-Fs. Positively selected CD271^+^ BM-MNCs as well as non-selected BM-MNCs were incubated for 14 days. Next, generated CFU-Fs were stained with Giemsa (*blue spots* on the *bottom* of tissue culture flasks) and then counted. Positively selected CD271^+^ BM-MNCs gave rise to a significantly higher number of CFU-Fs than non-selected BM-MNCs (P < 0.003). Results are persented as mean value ± standard error mean (n = 4). **b** Phenotype of CD271-MSCs was determined at the end of P2 and P4. Both types of MSCs studied revealed the same phenotype which is typcial for MSCs (negative for hematopoeitic markers and HLA-DR and positive for CD73, CD90, CD105 and HLA-Class I antigens). **c** Trilineage differentiation potential of both types of MSCs was evaluated by their culture in tissue-specific media for adipocytes, osteoblasts and chondrocytes. Confirmation of trilineage differentiation of MSCs was performed by staining with Oil Red O staining solution for adipocytes, SIGMA FAST™ BCIP/NBT (5-bromo-4-chloro-3-indolyl phosphate/nitro blue tetrazolium) tablets for osteoblasts and alcian blue solution for chondrocytes

Positively selected CD271^+^ BM-MNCs contained almost all progenitors for MSCs and therefore the number of CFU-Fs generated by using these cells was significantly higher than the number of generated CFU-Fs from non-selected BM-MNCs, as previously demonstrated [35] (Fig. 1a). *Ex vivo* expanded passage 2 and 4 CD271-MSCs were negative for the hematopoietic cell markers CD14, CD45, hematopoietic stem cell marker CD34 as well as class II HLA-antigens (HLA-DR). However, they expressed typical mesenchymal cell surface markers, such as CD73, CD90, CD105 and HLA class I molecules (Fig. 1b). In addition, flow cytometry analysis demonstrated that CD271 antigen is downregulated starting from passage 1 and is not detectable on the MSCs of either type during passage 2 (data not shown). Mesenchymal stromal cells generated through plastic adherence of unselected BM-MNCs (PA-MSCs) at passage 2 and 4 expressed the same typical MSC markers at the same level as CD271-MSCs, either. Culture of the expanded CD271-MSC in tissue-specific media demonstrated that these MSCs possess an equal potential as PA-MSCs to differentiate along adipogenic, osteogenic, and chondrogenic lineages (Fig. 1c).

### Evaluation of “wound closure” potential of MSCs

**Fig. 2:**
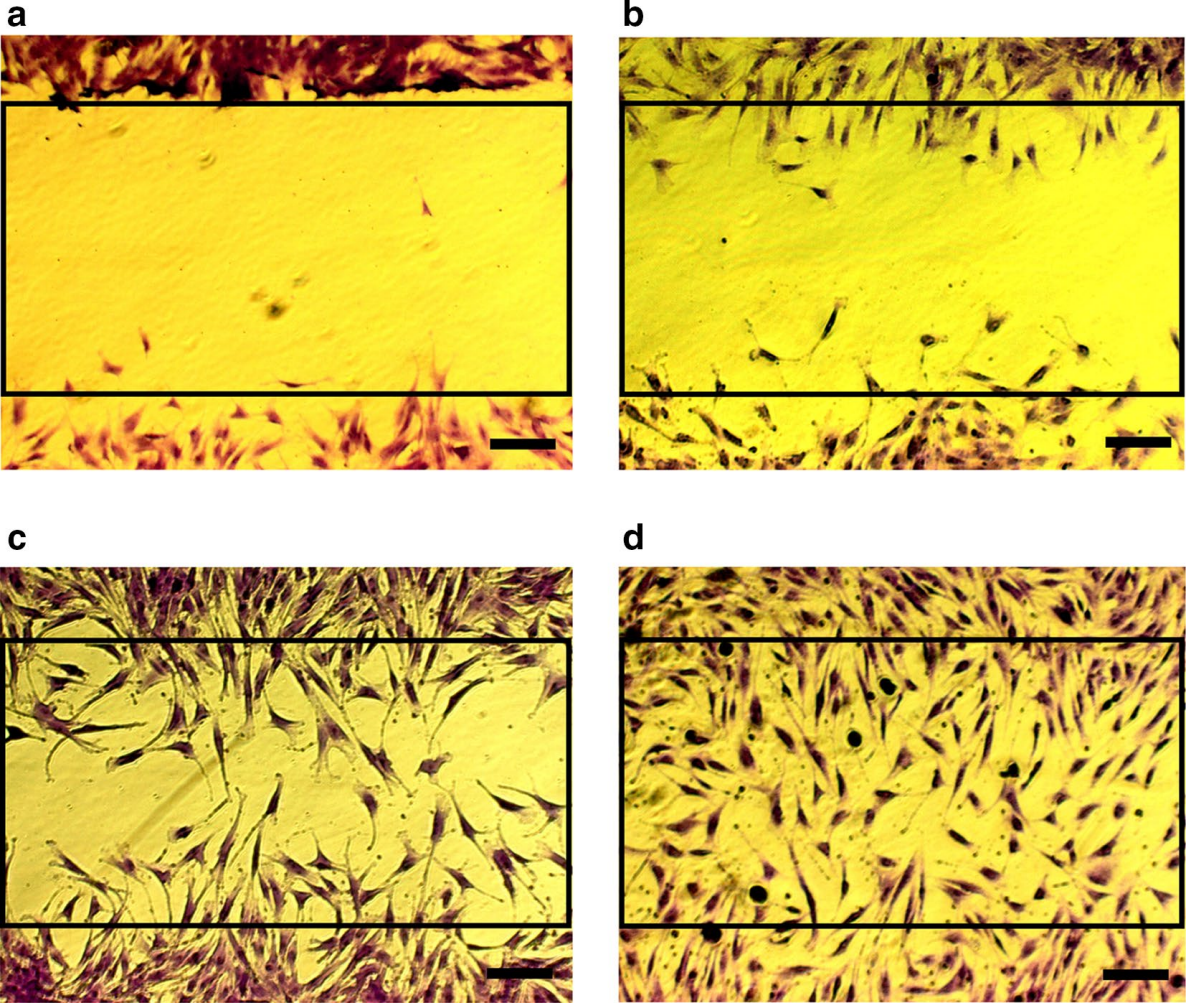
Schematic presentation of the estimation of wound healing. **a** Wound gap at the beginning of assay (0 h). **b** Start of CD271-MSC migration and wound closure after 6 h. **c** Migration and proliferation of CD271-MSCs 12 h after the start and **d** “Wound” closure 24 h later. To stain CD271-MSCs closing the wound, culture medium was removed and replaced with 400 µL of Cell Stain Solution for each well. Then the cells were incubated for 15 min at room temperature. After aspiration of staining solution, the cells were washed 3 times with deionized water and then were allowed to dry at room temperature (scale bars = 200 µm)

To analyse which types of MSCs (CD271-MSCs or PA-MSCs) are better in “wound healing”, we assessed this potential in both types of MSCs in the absence (DMEM supplemented with 1 % FBS only: control) and in the presence of GFs/Cytokines. For this purpose, we compared first the “wound healing” capacity between MSCs from different passages (P2 and P4) and thereafter we compared this potential between CD271-MSCs and PA-MSCs. Having in mind that wound healing process is affected by both migration and proliferation of cells, we compared it after 6 and 12 h (as a result of migration) and after 24 h (as a result of proliferation) (Fig. 2).

MSCs of both types were cultured 24 h until a monolayer formed at which time the inserts were removed to begin the wound healing assay. Cells were monitored under phase contrast (not shown) and followed by cell staining for determination of percent closure (0, 50, 75, and 100 %).

### The effect of passages on the “wound healing” potential of MSCs

**Fig. 3:**
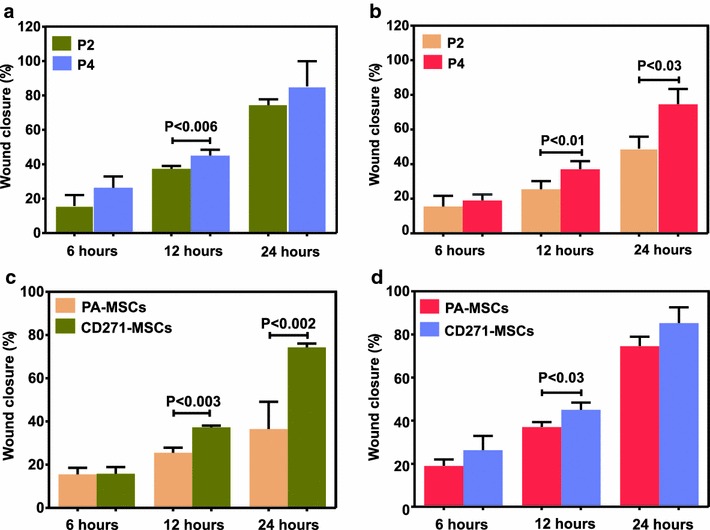
Wound healing potential of CD271-MSCs and PA-MSCs without exogenously added cytokines. **a** Comparison of wound healing potential of CD271-MSCs at passage 2 (P2) and 4 (P4). **b** Comparison of wound healing potential of PA-MSCs at passage 2 and 4. **c** Comparison of wound healing potential of PA-MSCs with that of CD271-MSCs at passage 2. **d** Comparison of wound healing potential of PA-MSCs with that of CD271-MSCs at passage 4. Results are presented as mean ± SD of 4 independently performed experiments. Statistical significance was evaluated by Student’s t test. Differences were considered significant when the P value was 0.05 or less

Before comparing “wound healing” capacity between CD271-MSCs and PA-MSCs, we asked whether the number of passages may affect this process. Comparative analysis of the data demonstrated that CD271-MSCs of passage 4 were significantly more effective than CD271-MSCs of passage 2 in closing the wound 12 h after starting the wound healing assay (P < 0.006). However, no differences in the “wound healing” potential between passage 2 and 4 of CD271-MSCs were observed six and 24 h after starting the assay (Fig. 3a). In contrast, passage 4 of PA-MSCs was significantly more efficient in closing the wound 12 and 24 h after starting the assay (P < 0.01 and P < 0.03, respectively) (Fig. 3b).

### CD271-MSCs possess a higher “wound healing” potential than PA-MSCs

In the current study we assessed the “wound healing” potential of CD271-MSCs and PA-MSCs both in the absence (DMEM supplemented with 1 % FCS only: control) and presence of GFs/Cytokines.

When compared “wound healing” capacity of CD271-MSCs to PA-MSCs in the absence (DMEM supplemented with 1 % FCS) of GFs/Cytokines, CD271-MSCs of passage 2 demonstrated a significantly higher potential to close the wound 12 and 24 h after initiation of the wound healing assay (P < 0.003 and P < 0.002, respectively). No differences were observed in the first 6 h of the wound closure (Fig. 3c). However, the “wound healing” potential of CD271-MSCs of passage 4 was significantly higher compared to PA-MSCs of the same passage only 12 h after initiation of the assay (P < 0.03) and no difference was observed for the time-points 6 and 24 h (Fig. 3d). Therefore, these data indicate that in particular CD271-MSCs of passage 2 are superior to PA-MSCs in the first 12 h of the healing process, demonstrating for the first time that these MSCs possess a higher migratory potential than PA-MSCs.

### Effect of various growth factors and cytokines on in vitro migration and proliferation potential of MSCs at different passages

**Fig. 4:**
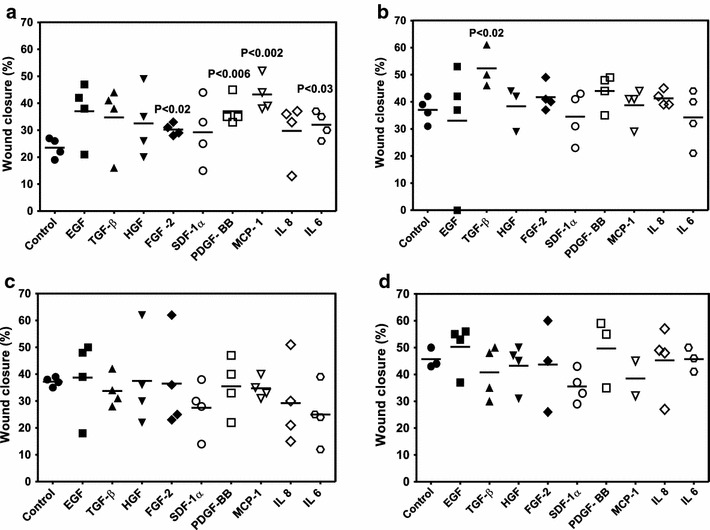
The effect of various growth factors and cytokines on wound healing potential of passage 2 and passage 4 of PA-MSCs and CD271-MSCs 12 h after the assay initiation. **a** The effect of GFs/cytokines on potential of passage 2 PA-MSCs in wound closure. **b** The effect of GFs/cytokines on potential of passage 4 PA-MSCs in wound closure. **c** The effect of GFs/cytokines on potential of passage 2 CD271-MSCs in wound closure. **d** The effect of GFs/cytokines on potential of passage 4 CD271-MSCs in wound closure. The effects of EGF (1 ng/ml), TGF-β (5 ng/ml), HGF (5 ng/ml), FGF-2 (20 ng/ml), SDF-1α (20 ng/ml), PDGF-BB (20 ng/ml), MCP-1 (20 ng/ml), IL-8 (20 ng/ml) and IL-6 (10 ng/ml) on wound closure were examined using CytoSelect™ 24-well Wound Healing Assay. Significant changes were assessed by using unpaired t test. Data are represented as mean ± SD of at least n = 2

It is well-known that different growth factors and cytokines are able to enhance MSCs migration [36, 37]. To date, however, there is no evidence on the effect of these molecules on migration potential of MSC-subsets. In this study we asked which growth factors and cytokines may affect migration of specific type of MSCs to “injured tissues” and their proliferation. Therefore, we compared the “wound healing” capacity of CD271-MSCs and PA-MSCs of passage 2 and 4 after their treatment with 10 different cytokines and growth factors. Our data show that compared to control, several growth factors significantly enhance “wound healing” capacity of PA-MSCs but not that of CD271-MSCs. The migration capacity of second passage PA-MSCs was significantly improved 12 h after treatment with FGF-2 (P < 0.02), PDGF-BB (P < 0.006), MCP-1 (P < 0.002) and IL-6 (P < 0.03) (Fig. 4a). TGF-β was the only cytokine that affected significantly the “wound healing” potential of passage 4 PA-MSCs 12 h after the treatment (P = 0.02) (Fig. 4b). These data are consistent with the findings of previous studies which demonstrated that MCP-1 [38] and PDGF-BB significantly enhanced migration of MSCs to the wounded site [39–41]. In addition, the effect of FGF-2 on MSC migration potential was also observed by other authors, who showed that MSCs are highly sensitive to FGF-2 [41, 42]. This is in line also with the observation of Schmidt et al. [42] who reported that migratory capacity of MSCs increases much more in presence of FGF-2 than IL-6. However, in contrast to other authors, EGF, HGF, TGF-beta, SDF and IL-8 did not significantly enhanced migration potential of PA-MSCs, as previously reported [43–46].

In contrast, neither growth factors nor cytokines were able to improve migration capacity of the second passage CD271-MSCs. Similarly to second passage, migration capacity and healing potential of passage 4 CD271-MSCs was not affected by a 12 h treatment with growth factors or cytokines (Fig. 4d). The greater capacity of CD271-MSCs to produce higher levels of cytokines than PA-MSCs [35] may account for this non-responsiveness of these cells to exogenously added cytokines/growth factors.

**Fig. 5:**
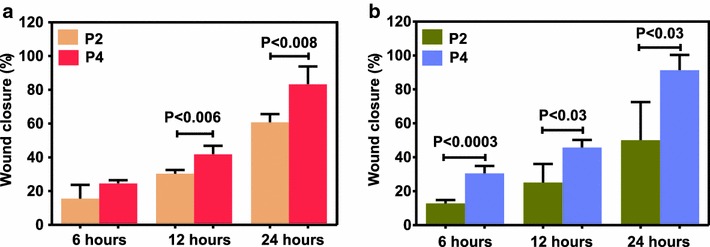
Effect of individual growth factors/cytokines on in vitro migration/proliferation capacity of mesenchymal stromal cells at different passages. **a** Effect of FGF-2 on different passages of PA-MSCs 6, 12 and 24 h after initiation of the wound assay. **b** Effect of IL-6 on different passages of CD271-MSCs 6, 12 and 24 h after initiation of the wound assay

To confirm that these effects of GFs/Cytokines are mediated via receptors, we used neutralizing PDGF-BB antibody and investigated migration of MSCs. We found that PA-MSCs migration stimulated by PDGF-BB (20 ng/ml) was inhibited by this neutralizing antibody at 2 µg/ml and this inhibition was significant on cells of passage 2 (P < 0.03) and passage 4 (P < 0.04) (data not shown), without abolishing the complete effect of PDGF-BB. This effect was consistent with the findings of Ozaki et al. [37], who observed the same effect in the migration experiments with rabbit MSCs. However, no changes were observed in the migratory capacity of CD271-MSCs after addition of the PDGF-BB antibody. Comparison of the effect of individual growth factors/cytokines on the “wound healing” capacity of MSCs at different passages demonstrated that only FGF-2 significantly increased “wound healing” potential of PA-MSCs of passage 4 compared to the cells of the second passage 12 and 24 h after initiation of the wound assay (P = 0.006 and P < 0.008, respectively) (Fig. 5a). In contrast, IL-6 was the cytokine which significantly improved “wound healing” potential of CD271-MSCs of fourth passage when compared to the CD271-MSCs of P2, 6, 12 and 24 h after initiation of the wound assay (P < 0.0003, P < 0.03 and P < 0.03, respectively) (Fig. 5b). This positive effect of IL-6 can be explained by the fact that CD271-MSCs produce significantly lower levels of IL-6 than PA-MSCs, as shown in our previous report [35].

## Conclusions

To the best of our knowledge, we show for the first time that during the wound closure, both CD271-MSCs and PA-MSCs of passage 4 were superior to MSCs of passage 2. In addition, the CD271-MSCs demonstrated a significantly higher “wound healing” potential than PA-MSCs, suggesting that they may be more effective in the treatment of the wounds.
